# Global research trends on exosomes in atherosclerosis: a bibliometric and scientometric analysis (2004–2025)

**DOI:** 10.3389/fcvm.2025.1700630

**Published:** 2026-01-07

**Authors:** Yubo Ren, Bomeng Zhao, Luo Lv, Wei Song, Zhu Wang, Zhihui Lu, Bao Li, Bin Yang

**Affiliations:** 1 Department of Cardiology, The Second Hospital of Shanxi Medical University, School of Medicine, Shanxi Medical University, Taiyuan, China; 2 Department of Respiratory and Critical Care Medicine, The Second Hospital of Shanxi Medical University, School of Medicine, Shanxi Medical University, Taiyuan, China; 3 Department of Cardiology, The First Hospital of Shanxi Medical University, School of Medicine, Shanxi Medical University, Taiyuan, China

**Keywords:** atherosclerosis, bibliometrics, exosomes, microRNAs, therapeutic potential

## Abstract

**Background:**

Exosomes play multifaceted roles in atherosclerosis, contributing to vascular inflammation, immune regulation, and tissue repair. This duality has drawn attention to their potential clinical utility, both as diagnostic indicators and as novel therapeutic avenues. However, despite the rapid expansion of research, a comprehensive scientometric overview of this field remains lacking.

**Methods:**

From 2004 to July 29, 2025, publications related to exosomes and atherosclerosis were screened in the Web of Science Core Collection (WoSCC) and Scopus. After deduplication, 1,179 records (964 articles, 215 reviews) were included. Bibliometric analyses were conducted using CiteSpace, VOSviewer, and the bibliometrix R package. Indicators examined included publication trends, global distribution, institutional and author contributions, co-citation patterns, and keyword evolution.

**Results:**

Publications on exosomes in atherosclerosis have risen sharply since 2015, consistent with Price's Law. China contributes the largest volume, while the United States shows stronger international collaboration. Central South University, Capital Medical University, and Harvard University rank among the most productive institutions. Key contributors include Elena Aikawa and Lijun Yuan. Major publication venues are the International Journal of Molecular Sciences, Frontiers in Cardiovascular Medicine, and Frontiers in Immunology. Keyword and co-citation mapping revealed three major research axes: RNA-mediated regulation, immune and endothelial interactions, and emerging translational applications such as engineered exosomes and diagnostic biomarkers.

**Conclusion:**

This bibliometric analysis characterizes global research development and shows a shift from descriptive studies toward mechanistic insight and translational innovation. These trends may facilitate future efforts in biomarker discovery, standardized exosome workflows, and therapeutic development.

## 1 Introduction

Atherosclerosis (AS) remains the primary cause of cardiovascular morbidity and mortality, despite significant advancements in the management of risk factors and improvements in revascularization techniques (1). Historically, AS was understood as the result of lipid deposition within the arterial walls (2); however, recent studies have revealed that it is a multifaceted, chronic immune-inflammatory disorder (3). This condition involves complex interactions between vascular and immune cells in the arterial wall, which contribute to endothelial dysfunction, vascular remodeling, and plaque formation (2).

Exosomes are small vesicles secreted by various cell types, and they play an essential role in intercellular communication by transporting microRNAs (miRs), proteins, lipids, and other molecular cargo to recipient cells (4). In the context of AS, exosomes modulate a range of pathological processes, including endothelial dysfunction, smooth muscle cell phenotypic alterations, foam cell formation in macrophages, vascular calcification, and thrombo-inflammatory responses (5, 6).

However, although research output in this field is growing rapidly, the literature lacks an integrated scientometric evaluation that maps its evolution, collaborative structure, and emerging thematic hotspots (5).

To address these gaps, this study conducted a bibliometric and scientometric analysis of global publications concerning exosomes in AS, with the aim of mapping the intellectual landscape, identifying emerging research trends, and highlighting key areas for future investigation. Using data from the Web of Science Core Collection and Scopus (2004–2025) and applying CiteSpace, VOSviewer, and Bibliometrix tools, we provide a structured overview of research development and thematic evolution. We offer a detailed assessment of global research activity in this field. This work aims to support future mechanistic studies, promote methodological standardization, and advance the clinical translation of exosome-based diagnostics and therapeutics in cardiovascular medicine.

## 2 Methods

### 2.1 Database and search strategy

While WoSCC is recognized for its stringent journal evaluation procedures and dependable citation tracking system, Scopus is valued for its wide disciplinary scope and sophisticated citation-analysis features that support interdisciplinary inquiry. Employing the two databases together allows researchers to obtain a more balanced and comprehensive evidence base, thereby enhancing the accuracy of bibliometric assessments and enabling deeper exploration of evolving research patterns and scholarly developments.

**Figure 1:**
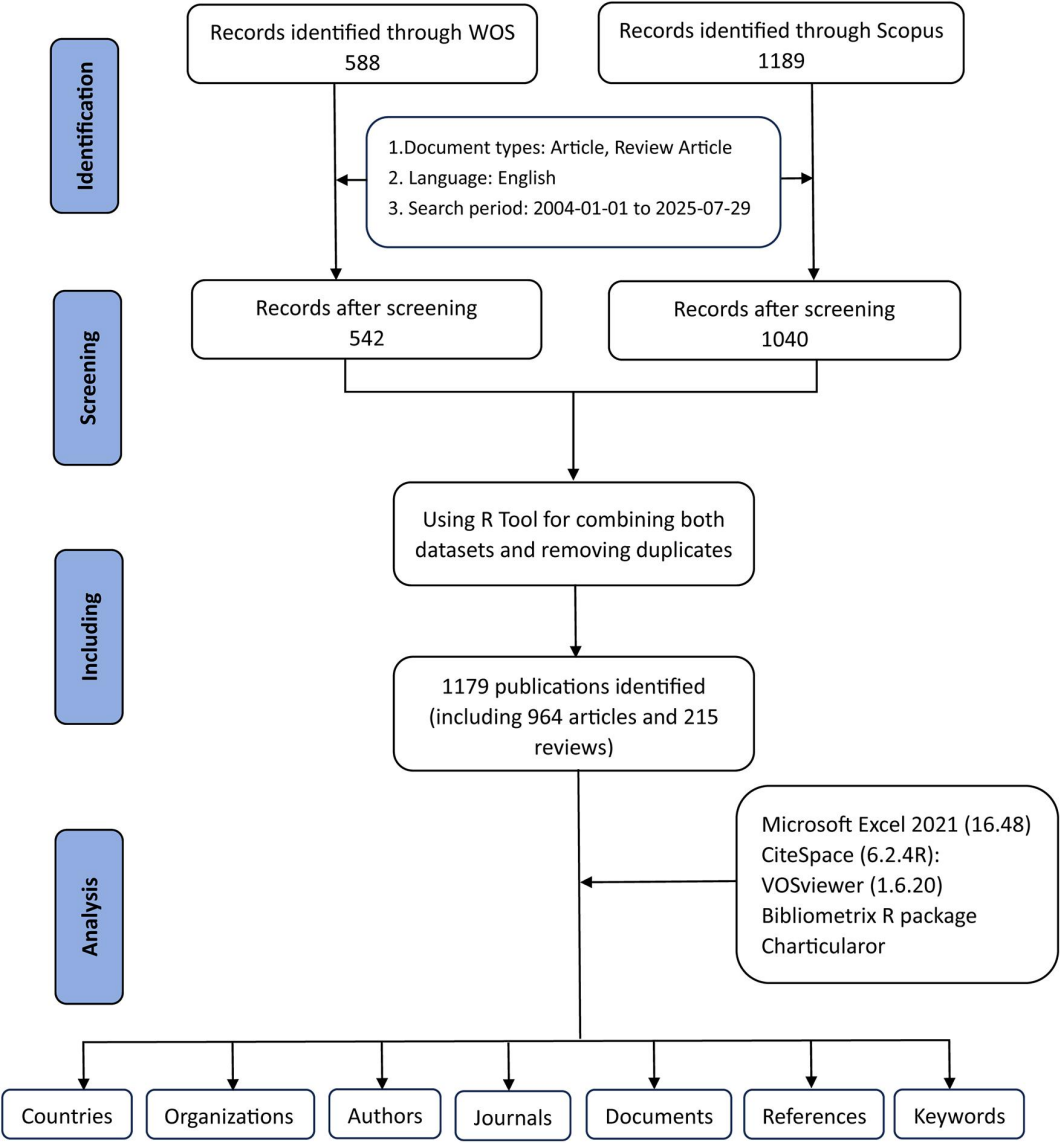
The flowchart for literature search, selection and analysis.

Figure 1 outlines the procedures for data retrieval and the criteria for excluding records. Bibliometric information was first collected by applying designated search terms in WoSCC and Scopus for the period from January 1, 2004, to July 29, 2025. A preliminary screening was then conducted using the publication titles, abstracts, and keywords. To define the search vocabulary, we consulted a range of earlier studies in the literature. Atherosclerosis-related terms = (“Atherosclerosis” OR “atherosclerosis” OR “atherogenesis”). Exosome-related terms = (“Exosome” OR “exosomal”). Finally, the two combined datasets yielded 588 documents from WoSCC and 1,189 documents from Scopus (Figure 1).

Exclusions were applied to remove Proceeding Papers, Corrections, Early Access articles, News Items, Book Chapters, Retractions, Reprints, Biographical Items, Book Reviews, Meeting Abstracts, Editorial Materials, and Letters. Only articles and reviews in English were retained. Additionally, the R tool was used to combine both datasets and removing duplicates. Ultimately, a total of 1,179 studies were included for further analysis, including 964 articles and 215 reviews, comprising 542 studies from WoSCC and 637 studies from Scopus (Figure 1).

### 2.2 Data analysis procedures

#### 2.2.1 Citespace

CiteSpace (6.2.4R, 64-bit Advanced Edition) was employed for the data analysis (7). The data collection period spanned from January 2004 to July 2025. The nodes analyzed included authors, institutions, and keywords. Only core analytical steps were retained in the main text, while detailed software parameters were streamlined for clarity. All records retrieved from WoSCC were saved as “full records and cited references” in plain text format.

#### 2.2.2 VOSviewer

VOSviewer (1.6.20), developed by CWTS at Leiden University, was utilized for processing the data. Full counting was used, with thresholds established based on analytical items to produce visual representations of collaborative networks (8).

#### 2.2.3 Bibliometrix

The bibliometrix R package (https://www.bibliometrix.org) (9), developed by Dr. Massimo Aria and Corrado Cuccurullo, was employed for historiographic analysis, tracking journal and author trends, and calculating bibliometric indicators such as g-index (10), h-index (11), number of citations (NC), and number of publications (NP).

#### 2.2.4 Other tools

Microsoft Excel 2021 (Version 16.48) was employed to organize the initial dataset. Bibliometric analyses of citation information were then conducted using the Online Analysis Platform of Literature Metrology (https://bibliometric.com/), which offers a straightforward and user-friendly analytical interface.

## 3 Results

### 3.1 Annual publication trends

**Figure 2:**
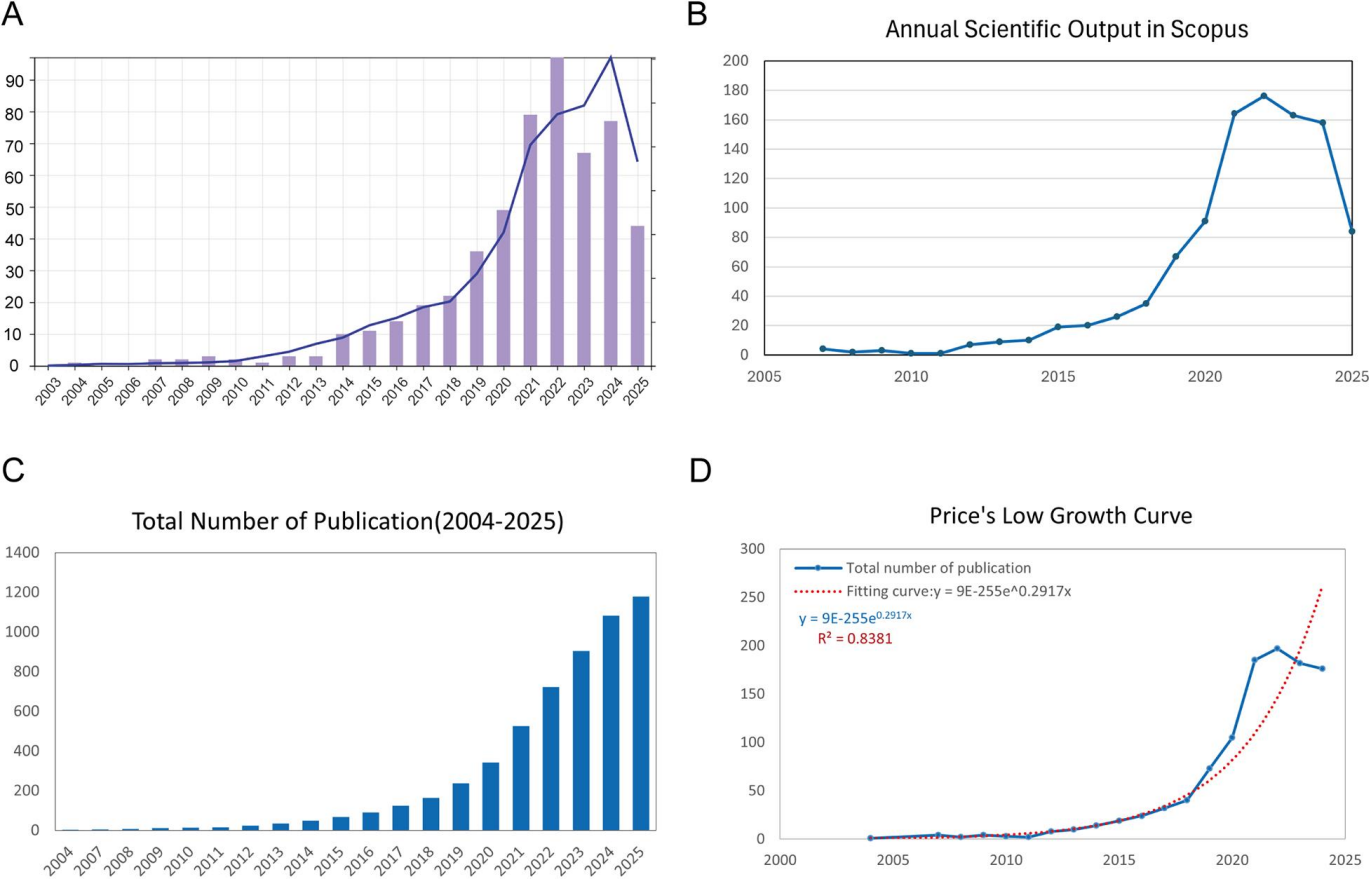
Overview of publications and citations on exosomes in atherosclerosis (2004–2025). **(A)** Annual trends in publication count (bars) and citation frequency (line) sourced from the WoSCC database. **(B)** Annual trends in publication count (bars) and citation frequency (line) sourced from the Scopus database. **(C)** Cumulative publication count over time, highlighting the overall expansion of the field. **(D)** Price's Law curve fitting analysis of cumulative publications, illustrating the exponential growth of exosome-related atherosclerosis research.

Our bibliometric analysis, spanning from January 2004 to July 2025, revealed a substantial increase in both the number of publications and citations in the field of exosomes and AS. A total of 1,179 documents were retrieved from the Scopus and WoSCC databases, with an annual growth rate of 24.4%, indicating a rapidly expanding research domain over the past two decades (Supplementary Table S1). Figure 2A shows that publication output remained low before 2015 but rose sharply thereafter, reflecting growing scientific interest. Citations exhibited a similar pattern, supporting the expanding influence of this field.

Figure 2B, sourced from Scopus, confirms the pattern observed in Figure 2A, showing a sharp increase in the number of publications after 2015, peaking in 2022. Although the number of publications in these two databases declined in 2025, it is important to note that this result is based on data up to July 29, 2025, and may not fully capture the trend for the entire year. We will monitor the full-year trends in subsequent updates. Figure 2C illustrates the cumulative publication count over time, demonstrating a clear exponential trajectory.

Importantly, Figure 2D confirms that the growth pattern aligns with Price's Law. The fitted model equation is *y* = 9E-255e^0.2917x^, with an *R*^2^ value of 0.8381, supporting the conclusion that exosome-related atherosclerosis research has entered a phase of accelerated scientific development.

### 3.2 Distributions of countries/regions

**Figure 3:**
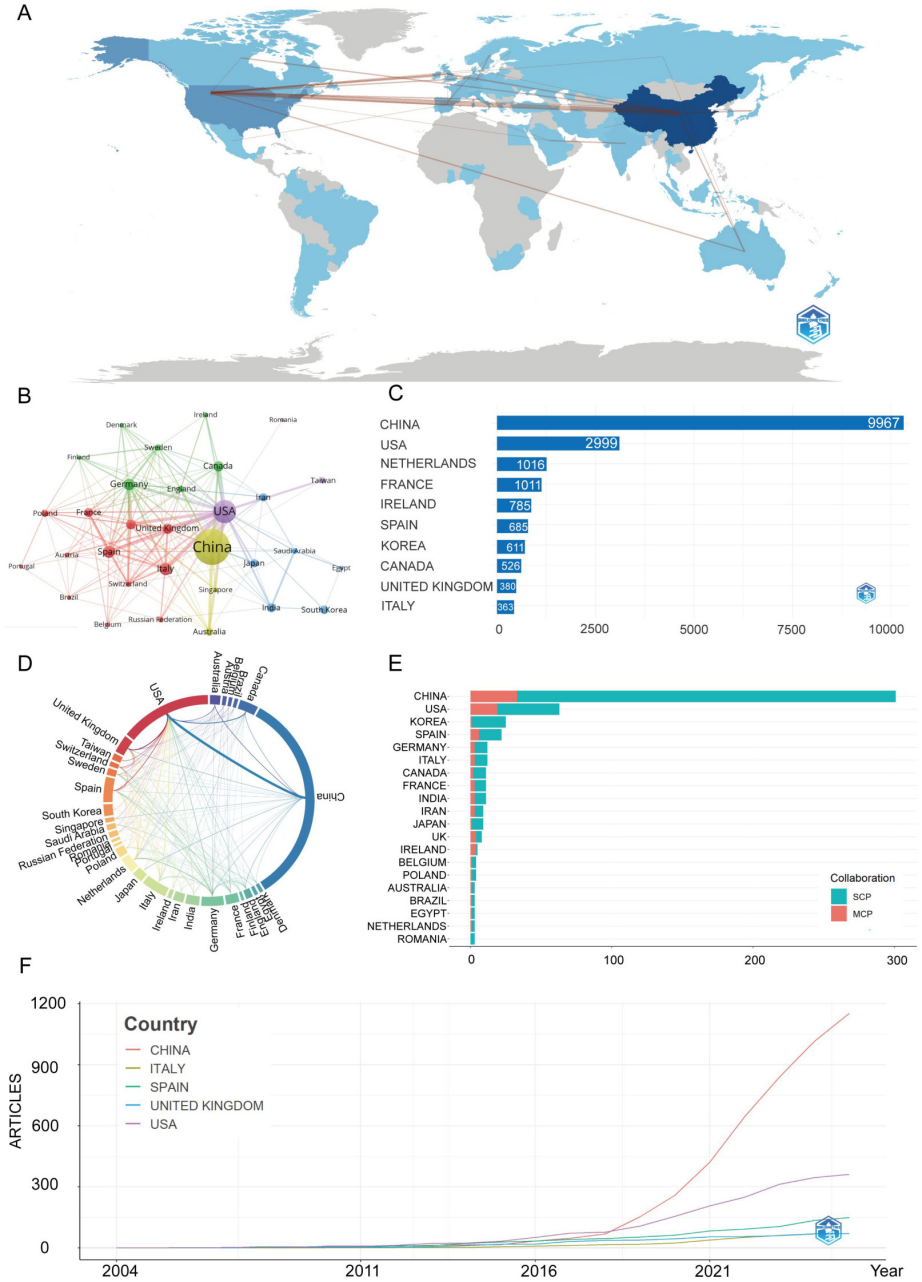
Global trends and collaboration networks in scientific publications on exosomes in atherosclerosis (2004–2025). **(A)** Country/Region Collaboration Map. Color depth represents the overall national collaboration intensity (the darker the color, the more active the collaboration), and line thickness represents the strength of bilateral collaboration (the thicker the line, the closer the cooperation between the two countries). **(B)** Country Clustering Analysis (network). Each node represents a country/region, with size proportional to publication volume. Edge thickness reflects co-authorship strength, and node color indicates collaboration clusters. Countries in the same color cluster collaborate more frequently, with spatial proximity indicating stronger links. **(C)** Top 10 Countries/Regions by Citation Impact. Horizontal bars display citation counts for each country/region's publications (labels indicate the count). **(D)** Network Map of National Research Output and Collaboration (chord diagram). Outer arcs represent countries/regions, with arc length showing overall collaborative output. Ribbons connect country pairs, with width indicating collaboration strength (number of co-authored papers). Ribbon colors match the originating arc. **(E)** Leading Countries by Publication Count and Collaboration Type. Stacked bars show the number of publications, split into single-country publications (SCP, teal) and multi-country publications (MCP, Salmon/red). The MCP proportion reflects international collaboration extent. **(F)** Top 5 Countries/Regions by Publications. Lines represent cumulative publications from 2004 to 2025, with year on the *x*-axis and cumulative count on the *y*-axis.

Figures 3A,B,D illustrate the global research network, with darker blue shades indicating stronger collaborative ties. In these visualizations, the core quantitative indicators in Figure 3A are calculated based on the number of co-published papers and weighted by the number of citations. The collaboration intensity in Figures 3B,D is measured using VOSviewer's total link strength, representing the volume of collaborative publications. The United States and China exhibit the most robust collaborative connections, forming dominant networks across North America, East Asia, and Europe. Other regions show comparatively limited participation.

In Figure 3C, citation performance mirrors production: China accrues the highest cumulative citations, signaling broad visibility and uptake. The United States ranks second, while European countries such as the Netherlands, France, Spain, and the United Kingdom contribute meaningfully.

Figure 3E shows China's leadership is driven mainly by single-country publications, reflecting strong domestic capacity. In contrast, the United States demonstrates a higher proportion of multi-country collaborations, consistent with a more globally integrated network.

Supplementary Table S2 and Figure 3F further illustrate that China shows the steepest growth curve, surpassing all other countries after 2018 and contributing the largest share of publications in recent years. The United States ranks second, with steady gains but a more moderate slope, while European contributors—particularly the United Kingdom, Spain, and Italy—exhibit smaller yet consistent increases.

### 3.3 Distribution by institutions

**Figure 4:**
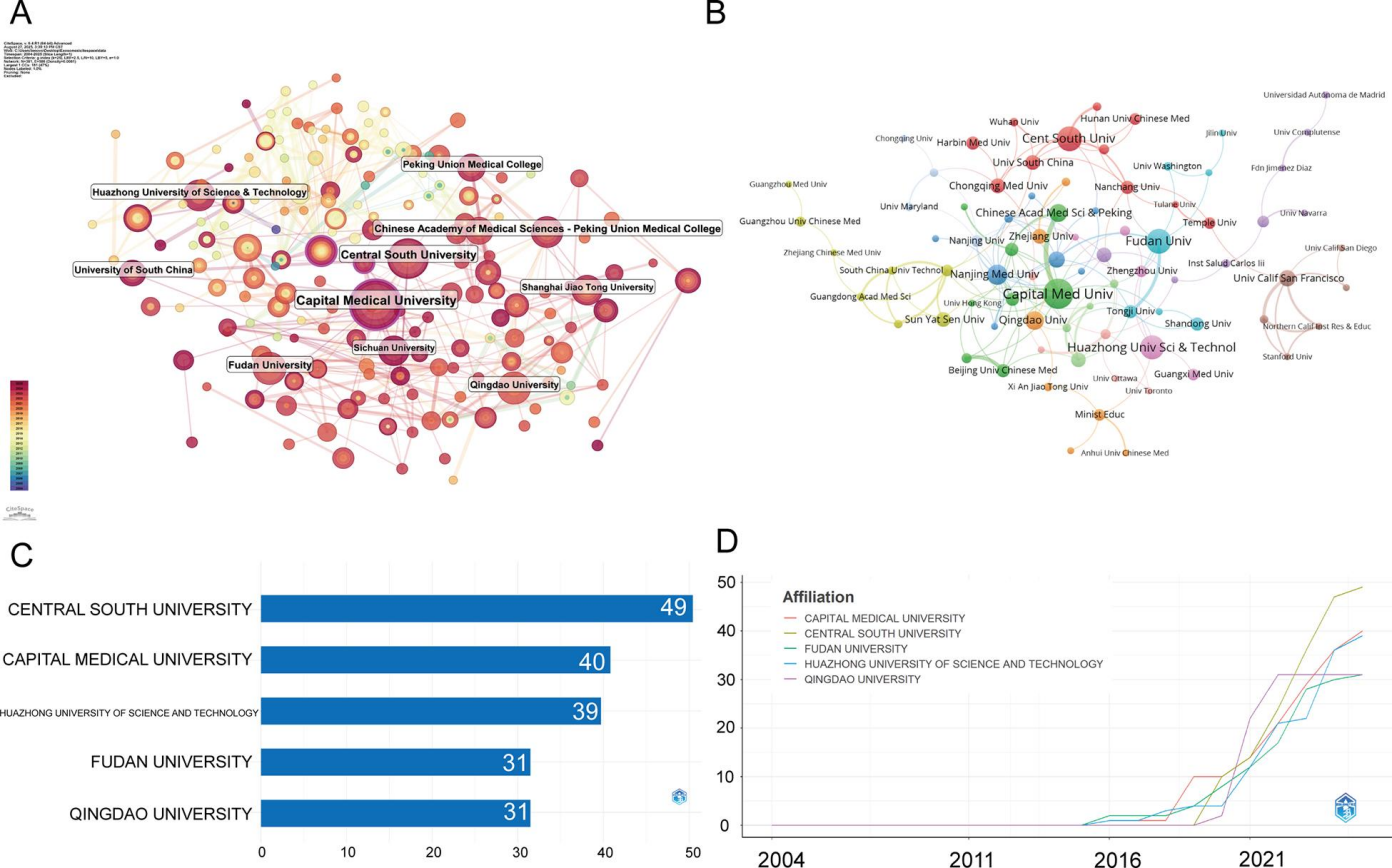
Institutional contributions and collaboration networks on exosomes in atherosclerosis (2004–2025). **(A)** Research institution network. Nodes represent institutions, with size indicating publication volume and line thickness showing collaboration strength. **(B)** Institutional collaboration network. Node size reflects collaboration extent, with colors indicating clusters based on region or affiliation. Line thickness represents cooperation intensity based on shared publications. **(C)** Top 5 Institutions by Citation Impact. **(D)** Institutional publication trends. The *x*-axis represents years, and the *y*-axis shows publication counts.

Figures 4A,B are the institutional co-occurrence diagram and the institutional cooperation network, respectively. Capital Medical University occupies the central position with the largest node size, indicating both high publication volume and frequent collaborations. Other major hubs include the Chinese Academy of Medical Sciences/Peking Union Medical College, Central South University, and Fudan University. These institutions form tightly connected clusters, reflecting strong domestic collaboration within China.

Figures 4C,D present a detailed overview of institutional contributions from January 2004 to July 2025. The top five institutions by publication output are Central South University (49 publications, 4.16%), Capital Medical University (40, 3.39%), Huazhong University of Science and Technology (39, 3.31%), Fudan University (31, 2.63%), and Qingdao University (31, 2.63%). Overall, institutional output is dominated by Chinese universities, with rapid growth observed after 2018.

### 3.4 Distributions of authors and co-cited authors

From 2004 to July 2025, a total of 6,836 authors contributed to 1,179 publications in the field of exosomes and AS, with an average of 6.94 co-authors per article, reflecting a highly collaborative research environment. Only 27 single-authored papers were identified, underscoring the dominance of teamwork in advancing this research area. The international co-authorship rate was only 8.74%, indicating limited cross-border collaboration (Supplementary Table S1). This outcome aligns with the patterns identified in the country-level analysis: although China contributes the largest number of publications, most are single-country studies, reflecting strong domestic research networks but relatively fewer international partnerships. The United States shows a higher proportion of multi-country publications, yet its smaller overall output does not offset the global imbalance, resulting in a modest international collaboration rate.

Such limited international cooperation may slow the integration of knowledge across regions and hinder the harmonization of research practices, including exosome-related technical methodologies. It may also affect the global influence of the field, because internationally co-authored studies typically achieve broader visibility and citation impact. Overall, the low rate suggests that research teams rely predominantly on national networks, which may restrict the diversity of perspectives and the pace of scientific convergence in this rapidly growing area.

**Figure 5:**
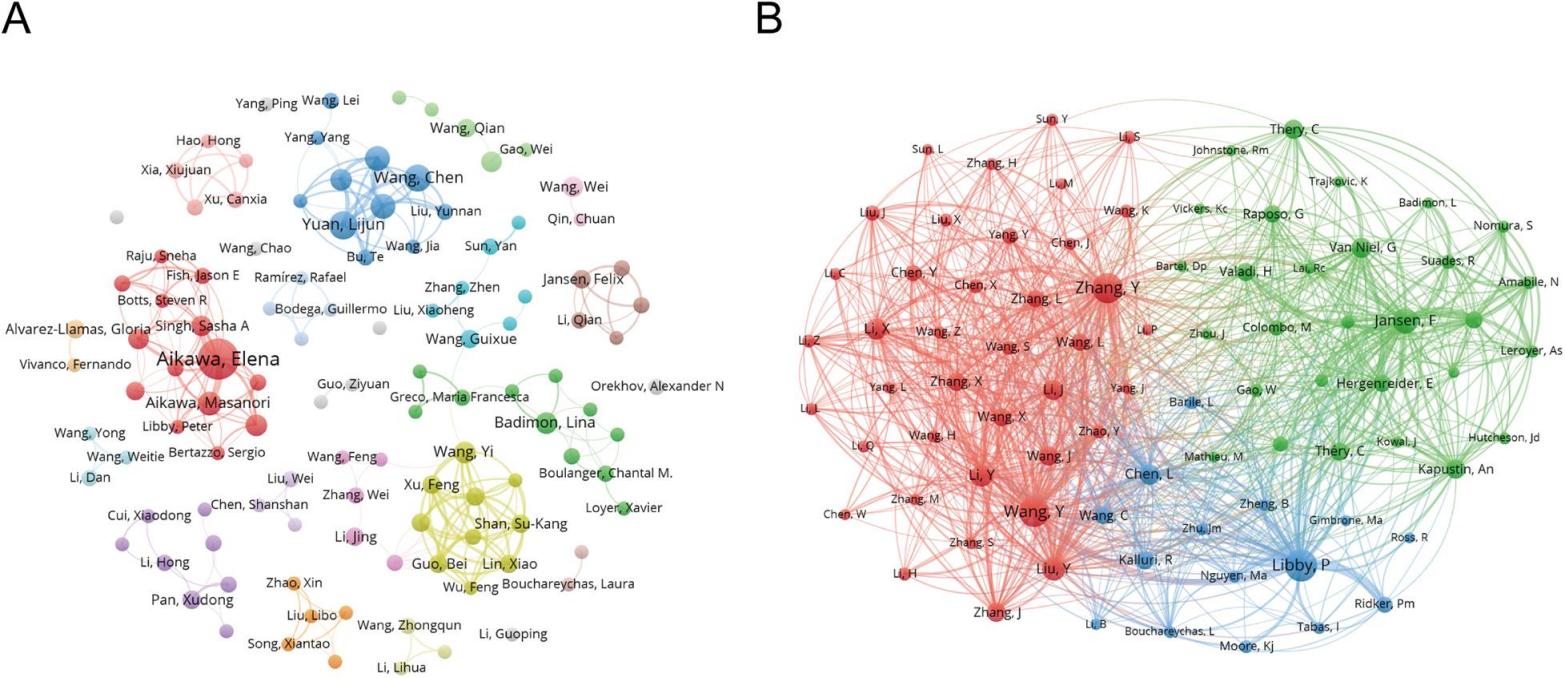
Co-authorship and Co-cited authorship networks on exosomes in atherosclerosis (2004–2025). **(A)** Co-authorship network: Nodes represent authors, with size reflecting publication count. Colors denote collaboration clusters. **(B)** Co-cited authorship network: Nodes, linked by joint publications, have edge thickness proportional to collaboration intensity. Clusters, indicated by color, reveal research groups.

In Figure 5A, the co-author network map reveals several distinct clusters, with Chinese scholars occupying the most prominent positions. Central nodes include researchers affiliated with leading institutions such as Central South University, Capital Medical University, and Fudan University. U.S. and European authors are often integrated through international partnerships.

Table 1 quantifies author influence, showcasing metrics like the g-index (10), h-index (11), and total citation counts (TC). Elena Aikawa and Masanori Aikawa remain leading contributors, while emerging scholars such as Lijun Yuan and Zhelong Li have shown rapid growth since 2021. Together, these authors shape both the historical foundation and recent expansion of the field.

**Table 1:** Top 10 authors on exosomes in atherosclerosis research (2004–2025).

Rank	Author	h_index	g_index	m_index	TC	NP	PY_start	Articles	Articles fractionalized
1	AIKAWA ELENA	17	21	1.308	1,731	21	2013	21	4.32
2	YUAN LIJUN	10	12	2	673	12	2021	12	1.59
3	LI ZHELONG	9	10	1.8	645	10	2021	10	1.40
4	WANG CHEN	9	11	1.8	505	11	2021	11	1.56
5	YANG GUODONG	8	10	1.6	414	10	2021	10	1.09
6	AIKAWA MASANORI	7	9	0.538	1,069	9	2013	9	0.68
7	BADIMON LINA	7	8	0.636	359	8	2015	8	1.70
8	GE JUNBO	7	7	0.7	473	7	2016	7	0.87
9	HUTCHESON JOSHUA D	7	8	0.538	737	8	2013	8	1.23
10	WANG YI	7	8	1	581	8	2019	8	0.70

TC, Total Citations; NP, Number of Publications; PY_start, Year of First Publication.

In Figure 5B, the co-citation network demonstrates clusters centered on influential scholars such as Elena Aikawa and Masanori Aikawa. Their studies form core intellectual nodes that are frequently co-cited. Additional co-cited authors, including Badimon and Junbo Ge, further reflect the field's mechanistic and translational research focus.

### 3.5 Journals and co-journals

**Figure 6:**
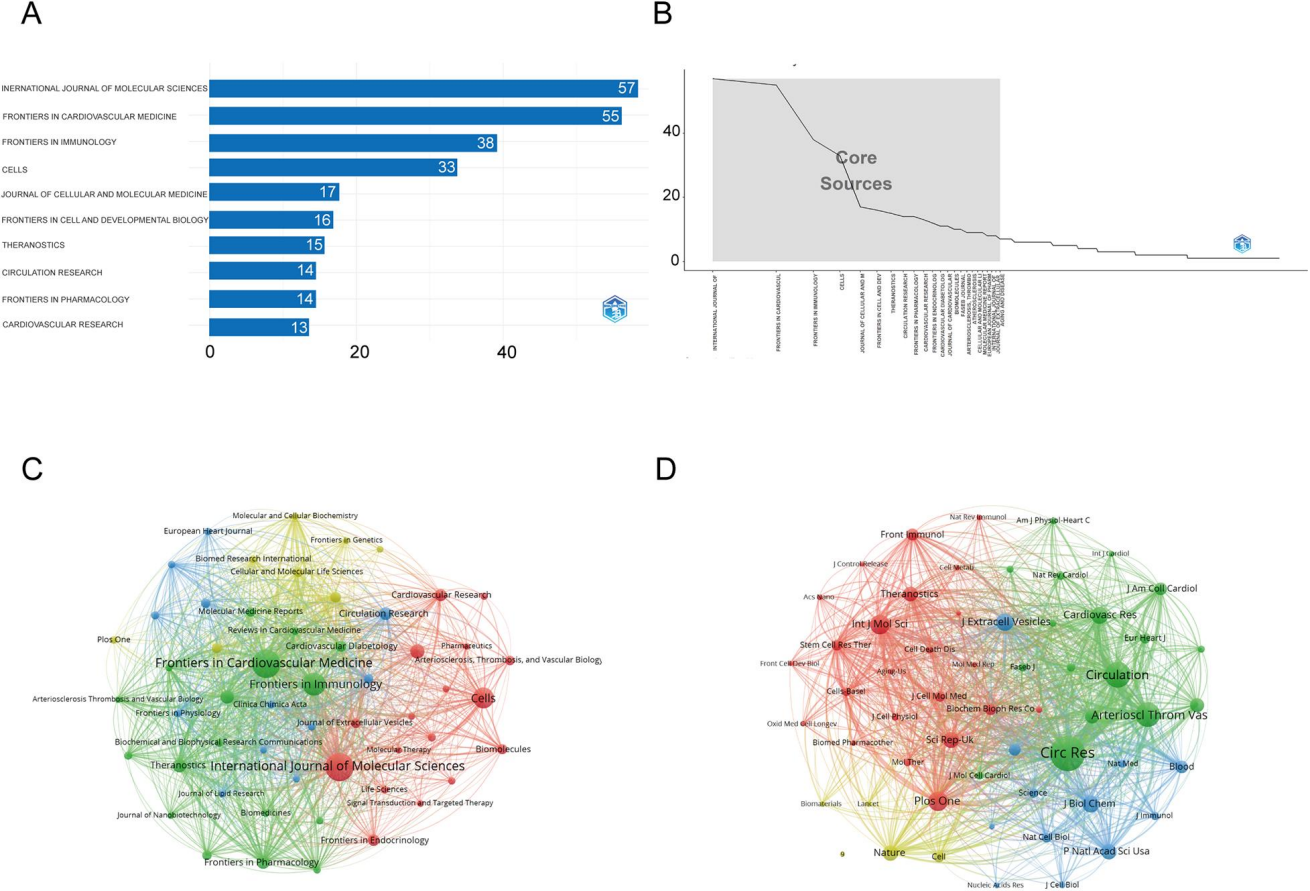
Comprehensive analysis of journals and citation networks related to exosomes in atherosclerosis (2004–2025). **(A)** Top journals by publication count: *X*-axis shows article numbers; *y*-axis lists journals. **(B)** Bradford's Law analysis: Identifies core journals based on publication distribution. **(C)** Journal citation network. Each node represents a journal. Node size is proportional to the number of publications, while links indicate citation relationships between journals. Line thickness reflects the strength of citation connections. Distinct colors (green, blue, red) denote clusters of journals that frequently cite each other, corresponding to thematic areas of research. **(D)** Journal co-citation network: Each node represents a journal, with size determined by co-citation frequency. Links indicate co-citation ties, with thicker lines reflecting stronger co-citation strength. Colors identify clusters of journals that are commonly co-cited.

Figure 6A shows that the ***International Journal of Molecular Sciences*** is the most influential source in exosome and atherosclerosis research, with 57 publications underscoring its central role in disseminating high-impact studies. It is closely followed by ***Frontiers in Cardiovascular Medicine*** (55 articles) and ***Frontiers in Immunology*** (38 articles). These journals account for a substantial proportion of the total research output in this area.

Table 2 further supports their academic importance: ***Frontiers in Immunology*** holds the highest h-index of 25 with more than 2,000 citations, while ***International Journal of Molecular Sciences*** demonstrates strong citation performance (h-index 22, 1,451 citations) and a Q1 JCR ranking. Likewise, ***Theranostics***, though publishing only 15 articles, exhibits a high impact factor (13.3, 2023 JCR), reflecting its significant influence relative to output.

**Table 2:** Top 10 influential journals on exosomes in atherosclerosis research (2004–2025).

Rank	Source	h_index	g_index	m_index	TC	NP	PY_start	IF	JCR
1	FRONTIERS IN IMMUNOLOGY	25	38	2.083	2,056	38	2014	5.9	Q1
2	INTERNATIONAL JOURNAL OF MOLECULAR SCIENCES	22	37	2.75	1,451	57	2018	4.9	Q1
3	FRONTIERS IN CARDIOVASCULAR MEDICINE	18	29	2	1,013	55	2017	2.9	Q1
4	CELLS	17	33	2.429	1,134	33	2019	5.1	Q2
5	THERANOSTICS	13	15	1.857	1,311	15	2019	13.3	Q1
6	FRONTIERS IN CELL AND DEVELOPMENTAL BIOLOGY	12	16	2	533	16	2020	4.3	Q1
7	FRONTIERS IN PHARMACOLOGY	11	14	1.222	456	14	2017	4.8	Q1
8	JOURNAL OF CELLULAR AND MOLECULAR MEDICINE	11	17	1.1	544	17	2016	4.2	Q2
9	CARDIOVASCULAR RESEARCH	10	13	0.714	1,699	13	2012	13.3	Q1
10	CIRCULATION RESEARCH	10	14	0.714	1,941	14	2012	20.8	Q1

IF, Impact Factor; JCR, Journal Citation Reports.

Figure 6B, in line with Bradford's Law, illustrates an uneven distribution of publications, where a limited number of journals—such as the ***International Journal of Molecular Sciences***, ***Frontiers in Cardiovascular Medicine***, and ***Frontiers in Immunology***—dominate the majority of research output. This indicates that research in this field is concentrated within a limited number of leading journals. Figure 6C, the journal co-occurrence map, demonstrates that publications on exosomes and atherosclerosis are concentrated in a limited number of core journals, with ***International Journal of Molecular Sciences***, ***Frontiers in Cardiovascular Medicine***, and ***Frontiers in Immunology*** forming the central nodes of scholarly communication. Figure 6D further shows that foundational journals such as ***Circulation Research***, ***Cardiovascular Research***, and ***Theranostics*** serve as major co-citation hubs, underscoring their influence on the development of exosome-related cardiovascular research.

### 3.6 References and articles

The field of exosome research in AS has grown rapidly over the past two decades, as shown in Supplementary Table S1. Between 2004 and July 2025, a total of 1,179 documents were published, drawing on 92,243 references. On average, documents are relatively recent, with a mean age of 3.75 years, and each receives 42.3 citations, reflecting both the novelty and impact of current research.

In Table 3, the most locally cited publications are led by Zhu JM (12) in *Theranostics* with 68 local citations and Li JB (13) in *Biochemical and Biophysical Research Communications* with 51 citations. These studies highlight key mechanistic insights into macrophage- and MSC-derived exosomes in AS. Other highly cited contributions, such as Boulanger (14) and Nguyen (15) further reinforce the central role of Extracellular vesicles in this field. Collectively, these publications represent key intellectual landmarks, anchoring the citation landscape of exosome and AS research.

**Table 3:** Top 20 most local cited publications based on bibliometrix analysis (2004–2025).

RANK	First Author	Year	Journal	Paper	DOI	Local Citations	Global Citations	LC/GC Ratio (%)	Normalized Local Citations	Normalized Global Citations
1	ZHU JM	2019	THERANOSTICS	Exosomes from nicotine-stimulated macrophages accelerate atherosclerosis through miR-21-3p/PTEN-mediated VSMC migration and proliferation	10.7150/thno.37357	68	314	21.66	12.32	3.56
2	LI JB	2019	BIOCHEM BIOPH RES CO	Exosomes derived from mesenchymal stem cells attenuate the progression of atherosclerosis in ApoE−/- mice via miR-let7 mediated infiltration and polarization of M2 macrophage	10.1016/j.bbrc.2019.02.005	51	179	28.49	9.24	2.03
3	BOULANGER CM	2017	NATURE REVIEW CARDIOLOGY	Extracellular vesicles in coronary artery disease	10.1038/nrcardio.2017.7	50	421	11.88	7.88	4.47
4	NGUYEN MA	2018	ARTERIOSCL THROM VAS	Extracellular Vesicles Secreted by Atherogenic Macrophages Transfer MicroRNA to Inhibit Cell Migration	10.1161/ATVBAHA.117.309795	50	189	26.46	7.52	2.14
5	GAO W	2016	J CELL MOL MED	Exosomes derived from mature dendritic cells increase endothelial inflammation and atherosclerosis via membrane TNF-alpha mediated NF-kappa B pathway	10.1111/jcmm.12923	47	237	19.83	4.39	2.40
6	BOUCHAREYCHAS L	2020	CELL REP	Macrophage Exosomes Resolve Atherosclerosis by Regulating Hematopoiesis and Inflammation via MicroRNA Cargo	10.1016/j.celrep.2020.107881	44	171	25.73	11.38	2.73
7	CHEN L	2017	PLOS ONE	Exosomal lncRNA GAS5 regulates the apoptosis of macrophages and vascular endothelial cells in atherosclerosis	10.1371/journal.pone.0185406	40	231	17.32	6.31	2.45
8	WANG C	2021	THERANOSTICS	Exosomes in atherosclerosis: Performers, bystanders, biomarkers, and therapeutic targets	10.7150/thno.56035	39	132	29.55	17.10	3.42
9	NIU CG	2016	J AM HEART ASSOC	Macrophage Foam Cell–Derived Extracellular Vesicles Promote Vascular Smooth Muscle Cell Migration and Adhesion	10.1161/JAHA.116.004099	37	138	26.81	3.46	1.40
10	LI JN	2017	THROMB RES	Thrombin-activated platelet-derived exosomes regulate endothelial cell expression of ICAM-1 via microRNA-223 during the thrombosis-inflammation response	10.1016/j.thromres.2017.04.016	35	156	22.44	5.52	1.66
11	XIE ZL	2018	J AM HEART ASSOC	Adipose-Derived Exosomes Exert Proatherogenic Effects by Regulating Macrophage Foam Cell Formation and Polarization	10.1161/JAHA.117.007442	33	151	21.85	4.96	1.71
12	ZHANG YJ	2010	MOL CELL	Secreted Monocytic miR-150 Enhances Targeted Endothelial Cell Migration	10.1016/j.molcel.2010.06.010	32	1013	3.16	2.13	1.39
13	ZHANG YG	2019	CELL CYCLE	Exosomes derived from oxLDL-stimulated macrophages induce neutrophil extracellular traps to drive atherosclerosis	10.1080/15384101.2019.1654797	32	97	32.99	5.80	1.10
14	XING XH	2020	AGING-US	Adipose-derived mesenchymal stem cells-derived exosome-mediated microRNA-342-5p protects endothelial cells against atherosclerosis	10.18632/aging.102857	32	93	34.41	8.28	1.49
15	TANG N	2016	FASEB J	Monocyte exosomes induce adhesion molecules and cytokines via activation of NF-*κ*B in endothelial cells	10.1096/fj.201600368RR	30	141	21.28	2.80	1.43
16	HULSMANS M	2013	CARDIOVASC RES	MicroRNA-21 controls neointimal lesion formation by regulating smooth muscle cell proliferation and apoptosis	10.1093/cvr/cvt161	28	291	9.62	4.24	2.49
17	NEW SE	2013	CIRC RES	Macrophage-Derived Matrix Vesicles: An Alternative Novel Mechanism for Microcalcification in Atherosclerotic Plaques	10.1161/CIRCRESAHA.113.301036	28	366	7.65	4.24	3.13
18	HE S	2018	SCAND J IMMUNOL	Endothelial extracellular vesicles modulate the macrophage phenotype: Potential implications in atherosclerosis	10.1111/sji.12648	28	107	26.17	4.21	1.21
19	WU GH	2020	ANGEW CHEM INT EDIT	Molecularly Engineered Macrophage-Derived Exosomes with Inflammation Tropism and Intrinsic Heme Biosynthesis for Atherosclerosis Treatment	10.1002/anie.201913700	28	212	13.21	7.24	3.39
20	HUANG CY	2018	MOL MED REP	Exosomal MALAT1 derived from oxidized low-density lipoprotein-treated endothelial cells promotes M2 macrophage polarization	10.3892/mmr.2018.8982	27	77	35.06	4.06	0.87

DOI, Digital Object Identifier.

**Figure 7:**
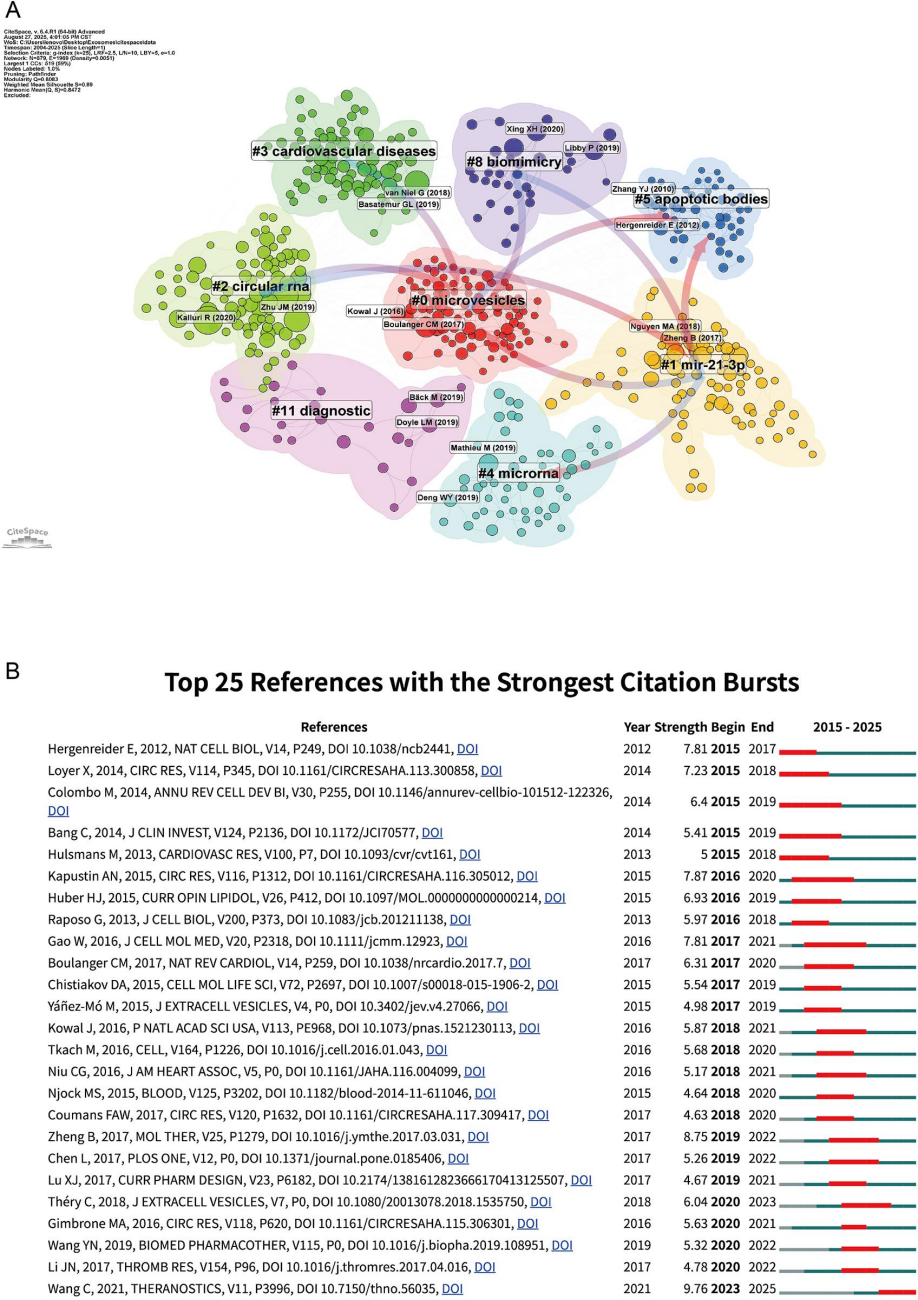
Network visualization of references and Top 25 references with strongest citation bursts on exosomes in atherosclerosis (2004–2025). **(A)** Clustered co-citation network based on keywords: Nodes, sized by citation frequency, are grouped by modularity with keyword labels for major clusters. Edge thickness reflects co-citation strength; colors highlight thematic diversity. **(B)** Top 25 References with Strongest Citation Bursts on Exosomes in Atherosclerosis: The burst timeline shows the periods during which these bursts occurred, with red segments indicating the time intervals when each reference received heightened attention.

Figure 7A depicts a well-structured co-citation landscape centered on Cluster 0 (“microvesicles”), which forms the core hub for foundational work on extracellular vesicles. From this core, several tightly connected clusters extend outward. Among them, Cluster 4 (“microRNA”) and Cluster 2 (“circular RNA”) highlight regulatory RNAs as major mechanistic focuses and link directly to Cluster 11 (“diagnostic”), underscoring the translational route from cargo characterization to biomarker development. Within this context, Cluster 1 (“miR-21-3p”) marks the convergence on specific effector miRNAs, bridging the “microRNA,” “microvesicles,” and “cardiovascular diseases” clusters and thus connecting mechanism with disease relevance. Along the historical axis, Cluster 5 (“apoptotic bodies”) captures early, frequently cited work that leads conceptually toward the current RNA-centered frameworks. Finally, Cluster 8 (“biomimicry”) represents the engineering and therapeutic strand, where insights from the mechanistic and diagnostic clusters are repurposed into exosome-mimetic, drug-loading, and targeted-delivery strategies.

Figure 7B lists the top 25 references with the strongest citation bursts. For example, Wang (5) show the most recent and intense influence, highlighting its role in shaping current research. In contrast, early bursts from Colombo (16) and Yáñez-Mó (17) exhibit sustained impact, reflecting their foundational contributions to extracellular vesicle biology. Théry (4) continues to guide the field's expansion toward immunological and mechanistic directions. Together, these bursts highlight both the historical foundations and evolving research hotspots in exosome–atherosclerosis studies.

### 3.7 Keywords analysis

The keyword analysis provides valuable insights into the thematic evolution of exosome research in AS. In total, 9,713 Keywords Plus and 2,510 author keywords were identified (Supplementary Table S1). This broad keyword base reflects the diversity and expansion of research topics in the field.

**Figure 8:**
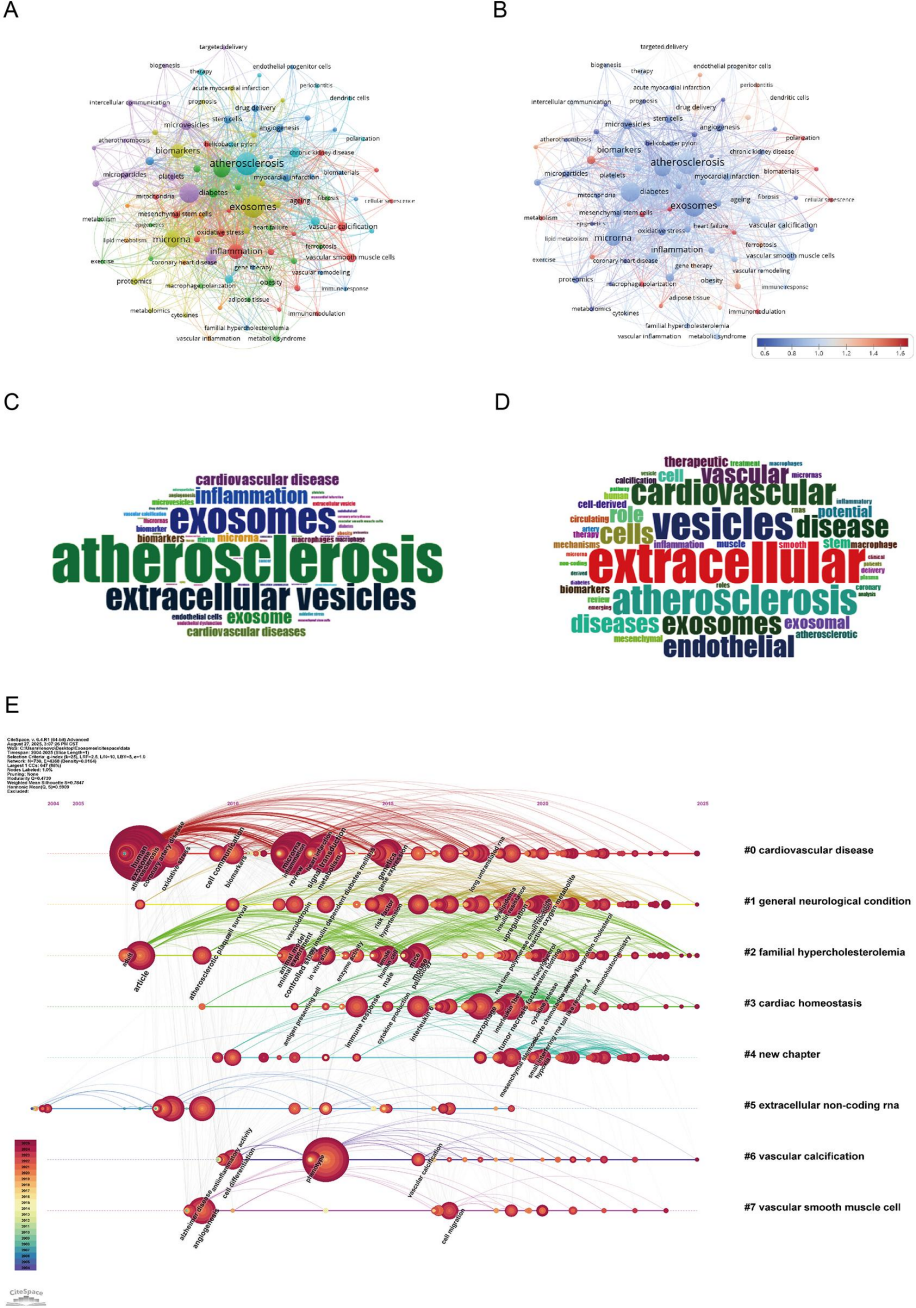
Keywords mapping of exosomes in atherosclerosis. **(A)** Keywords co-occurrence network: Each node represents a keyword, with size proportional to its frequency. Edges show co-occurrence relationships, with line thickness indicating co-occurrence strength. Colors represent thematic clusters identified by modularity analysis. **(B)** Keyword centrality network: Nodes represent keywords, sized by frequency and colored by betweenness centrality (redder nodes indicate higher centrality). Edges indicate co-occurrence relationships, highlighting pivotal keywords bridging clusters. **(C)** Author keywords word cloud: Word size reflects frequency, highlighting common author-assigned terms. **(D)** Title keywords word cloud: Word size indicates frequency, emphasizing key themes from titles. **(E)** Keyword timeline visualization: The *x*-axis shows publication years. Each horizontal line corresponds to a thematic cluster, labeled with a representative keyword. Nodes, sized by frequency, are positioned according to the year the keyword first appeared, visualizing topic emergence and evolution over time.

As illustrated in Figures 8A–D, the dominant research theme is clearly centered on “atherosclerosis” (382 occurrences, total link strength = 924), which appears as the most frequent and strongly linked keyword, reflecting its centrality in the field. Closely associated terms such as “extracellular vesicles” (263 occurrences, total link strength = 633) and “exosomes” (363 occurrences, total link strength = 843) underscore the pivotal role of vesicle biology in cardiovascular research. Mechanistic keywords including “inflammation” (129 occurrences, total link strength = 324) and “endothelial cells” (65 occurrences, total link strength = 177) highlight the growing attention to molecular pathways, particularly RNA-mediated regulation and endothelial dysfunction in disease progression (18, 19). The prominence of “biomarkers” (108 occurrences, total link strength = 273) and “oxidative stress” (24 occurrences, total link strength = 73) further demonstrates the translational orientation of the field, linking mechanistic studies with potential diagnostic and therapeutic applications (5). Keyword analysis shows that atherosclerosis continues to serve as the central focus, yet the field is expanding into mechanistic discovery, biomarker development, and translational applications, establishing exosome research as an emerging frontier in cardiovascular medicine.

As shown in the keyword timeline (Figure 8E), the evolution of research on exosomes in AS demonstrates clear thematic and temporal progression from 2004 to July 2025. The earliest cluster, “cardiovascular disease” (Cluster 0), emerged as a foundational theme and has remained consistently relevant, reflecting the central role of exosome-mediated mechanisms in cardiovascular pathology. Around the mid-2010s, attention expanded to more specific disease contexts such as “general neurological condition” (Cluster 1) and “familial hypercholesterolemia” (Cluster 2), illustrating the diversification of research into comorbidities and genetic predispositions that influence AS progression.

Subsequently, clusters such as “cardiac homeostasis” (Cluster 3) and “extracellular non-coding RNA” (Cluster 5) became increasingly prominent after 2018, highlighting the mechanistic focus on immune regulation, endothelial dysfunction, and RNA-based signaling pathways. These terms emphasize the growing interest in RNA-mediated regulation of gene expression and its implications for vascular remodeling and inflammatory control.

Overall, keyword evolution demonstrates a transition from broad cardiovascular concepts to mechanistic pathways and translational applications, underscoring the growing depth and clinical relevance of exosome research in AS.

## 4 Discussion

Exosomes interact with various vascular processes, influencing immune cell behavior, endothelial function, smooth muscle cell growth, and blood vessel remodeling. By carrying microRNAs and proteins, exosomes can either promote inflammation or aid in repair, depending on the disease stage. Additionally, exosomes show promise as drug delivery systems, offering targeted treatments and regenerative potential. As we learn more about their biological functions, bioengineered exosomes could lead to more effective, personalized therapies for cardiovascular diseases like AS.

### 4.1 Research highlights

Keyword analysis and co-citation mapping jointly reveal the five most prominent research directions in the current study of exosomes in AS: exosomes in immune cell interactions, exosomal impact on endothelial dysfunction, exosomes in vascular smooth muscle cell proliferation and migration, platelet exosomes in atherosclerosis, exosomes in atherosclerosis: role in vascular repair and remodeling, and exosomal therapeutic potential in atherosclerosis. The following subsection provides a focused synthesis and integrated discussion of these hotspots.

#### 4.1.1 Emerging roles of exosomes in immune cell interactions

Immune regulation represents one of the central mechanisms by which exosomes participate in AS.

Accumulating evidence indicates that macrophage-derived exosomes serve as pivotal signaling vectors that sculpt the local inflammatory milieu; key miRNAs—most notably miR-146a and miR-155—modulate NF-κB and JAK/STAT pathway activity, thereby governing inflammatory amplitude, cellular motility, and chemotactic responses within atherosclerotic lesions (15).

Exosomes released by macrophages often carry specific microRNAs, such as miR-146a and miR-155-5p, each exerting distinct effects on immune regulation (20). MiR-146a alleviates inflammation and slows AS progression by dampening NF-κB signaling and down-regulating TNF-α, IL-1β and other pro-inflammatory mediators (21). However, under pathological conditions such as impaired endothelial repair or sustained macrophage retention, miR-146a can switch to a pro-atherogenic role by suppressing endothelial cell migration and promoting macrophage persistence within the plaque (15).

In contrast, miR-155-5p facilitates immune activation by inhibiting negative regulators of the immune response, such as suppressor of cytokine signaling 1(SOCS1) (22). Conversely, miR-155-5p targets SOCS1 to activate the JAK2/STAT3 axis, thereby amplifying the production of IL-6, IL-12 and other inflammatory mediators, precipitating endothelial senescence and exacerbating vascular inflammation (23, 24). This sustained immunoinflammatory response accelerates atherosclerotic progression and destabilizes plaques, while circulating miR-155-5p is increasingly recognized as a diagnostic and prognostic biomarker for cardiovascular disease (25, 26).

Although the detailed molecular mechanisms remain partially unresolved, it is now established that exosomes constitute an intercellular communication network among immune cells and operate critically during the initiation, amplification, and maintenance phases of atherosclerosis. Consequently, their immunomodulatory properties are increasingly recognized as promising therapeutic targets.

#### 4.1.2 Exosomal impact on endothelial dysfunction

Recent studies highlight endothelial-derived exosomes as important mediators of cell-to-cell communication, particularly through miRNAs such as miR-126 and miR-210 (18). MiR-126 has been closely linked to endothelial stability, in part through the regulation of pro-inflammatory mediators like the CXCL12-CXCR4 axis, which influences immune cell recruitment and vascular inflammation (27). It is also thought to strengthen barrier properties, stabilize intercellular junctions, and modulate vascular permeability, thereby safeguarding endothelial integrity. In contrast, miR-210 is predominantly induced under hypoxic conditions, a feature of atherosclerotic lesions. By modulating pathways such as vascular endothelial growth factor (VEGF), it promotes angiogenesis and reduces apoptosis, while also influencing smooth muscle cell migration and differentiation, changes that support fibrous cap formation and enhance plaque stability (28).

Endothelial-derived exosomal miRNAs exert context-specific effects: although they frequently support endothelial integrity and facilitate vascular repair, under pathological conditions they can also drive maladaptive remodeling and accelerate plaque development (12, 29, 30). In our analysis, the frequent clustering of the keywords reinforces this line of research, suggesting a decisive role for endothelial dysfunction in the early phases of AS.

#### 4.1.3 Exosomes in vascular smooth muscle cell proliferation and migration

Recent studies indicate that Vascular Smooth Muscle Cell-derived exosomes (VSMC-derived exosomes) represent a central signaling hub in atherosclerosis, linking calcification, angiogenic activity, and phenotypic modulation within a coherent pathogenic network. Rather than acting through isolated mechanisms, these exosomes coordinate VSMC transition, extracellular matrix remodeling, and endothelial responses through their microRNA cargo, including miR-155-5p (31–34). Their involvement in calcium deposition and neovascularization aligns with features of plaque growth and vulnerability, positioning VSMC-derived exosomes as key contributors to the evolution of unstable lesions (30, 33). Overall, the emerging literature highlights these vesicles not only as mediators of VSMC proliferation and migration but as integrators of multiple processes that shape plaque development across different disease stages.

#### 4.1.4 Platelet-derived exosomes in atherosclerosis

Platelet-derived exosomes have gained increasing attention in AS research because they sit at the interface of thrombosis and vascular inflammation. These vesicles participate in early plaque formation and contribute to the modulation of plaque stability by delivering microRNAs implicated in vascular injury and endothelial responses (35). Among them, miR-223 plays a representative role by altering platelet–endothelial communication through ICAM-1 regulation (36), illustrating how platelet-derived exosomes integrate inflammatory signaling with thrombotic activity. Their functional effects vary with the local microenvironment, exhibiting either protective or pro-atherogenic patterns across different disease stages (37). In addition, accumulating evidence suggests that these exosomes support endothelial repair and attenuate vascular injury, underscoring their potential therapeutic relevance (35). Overall, platelet-derived exosomes are emerging as key mediators at the inflammation–thrombosis intersection, linking platelet activation with vascular dysfunction and shaping atherosclerotic disease progression.

#### 4.1.5 Exosomes as regulators of vascular repair and remodeling

In AS, exosomes play a key role: they can exacerbate inflammation and disease progression, while also contributing to vascular repair and remodeling.

Within the progression of AS, exosomes participate not only in inflammatory amplification but also in vascular repair, forming a dual regulator*y* axis that becomes particularly relevant in advanced lesions. Among the reparative vesicles, endothelial progenitor cell–derived exosomes (EPC-exosomes) have received considerable attention because they promote endothelial resilience through coordinated effects on survival pathways, cytoprotection, and functional recovery (38–40). Rather than acting through isolated mechanisms, EPC-exosomes integrate pro-repair signals—including ACE2/Ang-(1–7)/Mas activation and microRNA transfer—into a broader response that supports endothelial restoration and vascular elasticity. Their efficacy, however, varies with the local inflammatory and hemodynamic milieu, highlighting the context dependence of exosome-mediated repair (41). Overall, current findings suggest that exosome-driven vascular remodeling represents an emerging translational direction, consolidating diverse reparative signals into a potential therapeutic strategy aimed at stabilizing the atherosclerotic vasculature.

#### 4.1.6 Therapeutic potential and translational prospects of exosomes in atherosclerosis

Exosomes are emerging as promising therapeutic platforms in AS, where their lipid bilayer and low immunogenicity enable targeted drug delivery to diseased vascular sites (42). In addition to this delivery capacity, they naturally carry proteins, lipids, and RNAs that regulate cellular behavior and disease progression, underscoring their value as crucial mediators in the treatment of AS, owing to their ability to support intercellular communication and to transport bioactive molecules that shape disease progression (43). Exosomal X26nt derived from vascular smooth muscle cells regulates phenotypic switching and oxidative stress by reducing ER stress and enhancing SOD1 expression, indicating that exosomal RNAs such as X26nt may represent promising therapeutic targets (43). They further exhibit a dual role in AS by amplifying inflammation through pro-inflammatory signaling and oxidative stress while simultaneously promoting anti-inflammatory responses, a complexity that highlights their central involvement in disease progression and regulation (44). Additionally, plasma exosomal miR-30e and miR-92a, by targeting ATP-binding cassette transporter A1 (ABCA1), are upregulated in AS, providing new biomarkers for the clinical diagnosis and treatment of coronary AS (45).

In recent years, bioengineered exosomes have shown significant potential in the treatment of AS. For example, macrophage-derived exosomes carrying 5-aminoacetylpropionyl hexyl ester have demonstrated significant therapeutic effects on AS (46). In addition, regulating the release of exosomal microRNAs has been proposed to dampen immune activation and limit foam cell formation. Advances in bioengineering further expand the therapeutic landscape of exosomes, allowing surface functionalization with targeting ligands and the development of more efficient drug-loading technologies (42). Through the artificial modification of exosomes, systems such as thermosensitive liposomal mixed nanocarriers and membrane-coated nanoparticles are expected to overcome challenges such as low drug loading capacity and suboptimal delivery efficiency of exosomes (47). Compared with synthetic nanoparticles, exosomes combine inherent biocompatibility with superior barrier penetration, underscoring their promise as next-generation candidates for cardiovascular therapy (48).

Exosomes are positioned as a multifaceted therapeutic platform, functioning both as carriers of bioactive agents and as modulators of the vascular microenvironment, with the potential to attenuate atherosclerotic progression. In the future, exosome-like nanoparticle therapy is expected to provide a novel and effective treatment option for patients with AS.

### 4.2 Limitations

Several limitations of this study should be acknowledged. To begin with, the analysis relied mainly on the Web of Science Core Collection and Scopus. Although these are widely used sources, the omission of databases such as PubMed or Embase means that some relevant work might not have been captured, raising the possibility of database-related bias. It should also be acknowledged that we confined our search to English-language articles and reviews, which inevitably narrows the global perspective by overlooking non-English contributions. In addition, bibliometric indicators, including citation counts and the h-index, are shaped by factors such as database coverage, self-citation practices, and the passage of time; these influences need to be borne in mind when interpreting research impact. The software tools applied in this work, namely CiteSpace, VOSviewer, and Bibliometrix, are valuable for mapping and visualization but cannot by themselves reflect the quality or rigor of individual studies, which calls for complementary domain expertise. Finally, our analysis emphasized quantitative mapping rather than detailed biological or clinical interpretation. To translate insights from exosome research into practice, further systematic reviews and experimental validation will be necessary.

While the study has certain constraints, drawing on two widely recognized databases together with several bibliometric tools lends robustness to the analysis and helps outline major global research trends. Looking ahead, a broader approach that brings in other databases, non-English literature, and evidence from clinical trials could offer a more nuanced and comprehensive understanding of how exosomes contribute to AS.

## 5 Conclusion

Exosomes are increasingly recognized as central mediators in the pathogenesis of AS, influencing immune activation, endothelial dysfunction, vascular smooth muscle remodeling, and plaque stability. Our bibliometric analysis shows that research in this domain has grown exponentially over the past two decades, with a pronounced surge after 2015, reflecting the shift from descriptive studies to mechanistic and translational investigations. The emergence of keywords such as *inflammation*, *biomarkers*, *vascular calcification*, and *RNA-mediated regulation* highlights how the field is converging on molecular pathways with direct implications for diagnosis and therapy.

The results of this analysis highlight both the promise and the obstacles that remain in the field. Exosomes are emerging as minimally invasive biomarkers and as therapeutic carriers with the potential to target vascular lesions in a highly specific way. Yet their broader application is still constrained by inconsistent isolation protocols, limited international collaboration, and a gap between experimental progress and clinical translation.

Research on exosomes in AS is now shifting from descriptive studies toward precision medicine and regenerative approaches. Greater use of standardized methodologies, incorporation of multi-omics technologies, and advances in bioengineering for exosome modification could help overcome existing barriers and speed progress toward clinical application. If these developments continue, exosome-based diagnostics and therapies may redefine cardiovascular care by enabling earlier diagnosis, improved risk stratification, and novel strategies for vascular repair.

## Data Availability

The raw datasets analyzed in this study were obtained from Web of Science and Scopus via institutional subscriptions. Due to copyright restrictions, these datasets are not publicly available. Processed data (e.g., extracted keywords, citation networks) are available upon reasonable request. Requests to access these datasets should be directed to Yubo Ren, yuboren2002@163.com.
