# Supplementary material for: Global research trends on exosomes in atherosclerosis: a bibliometric and scientometric analysis (2004–2025)
Source: Front Cardiovasc Med. 2026 Jan 7;12:1700630. doi: 10.3389/fcvm.2025.1700630 (PMC12819766; doi:10.3389/fcvm.2025.1700630)
Supplement: Supplementary file 1 [file Datasheet1.docx]

**Supplementary Table S1. Bibliometric Overview of Exosomes in Atherosclerosis (2004–2025)**

| **Description** | **Results** |
| --- | --- |
| MAIN INFORMATION ABOUT DATA |  |
| Timespan | 2004:2025 |
| Sources (Journals, Books, etc) | 485 |
| Documents | 1179 |
| Annual Growth Rate % | 24.4 |
| Document Average Age | 3.75 |
| Average citations per doc | 42.3 |
| References | 92243 |
| DOCUMENT CONTENTS |  |
| Keywords Plus (ID) | 9713 |
| Author's Keywords (DE) | 2510 |
| AUTHORS |  |
| Authors | 6836 |
| Authors of single-authored docs | 27 |
| AUTHORS COLLABORATION |  |
| Single-authored docs | 27 |
| Co-Authors per Doc | 6.94 |
| International co-authorships % | 8.736 |
| DOCUMENT TYPES |  |
| article | 964 |
| review | 215 |

Co-Authors per Doc: Average number of authors per document (total appearances / documents, distinct from the total unique authors. )

**Supplementary Table S2. Most Relevant Countries by Corresponding Author Contributions**

| Rank | **Country** | **Articles** | **Articles %** | **SCP** | **MCP** | **MCP %** |
| --- | --- | --- | --- | --- | --- | --- |
| 1 | CHINA | 301 | 25.5 | 268 | 33 | 11 |
| 2 | USA | 63 | 5.3 | 44 | 19 | 30.2 |
| 3 | KOREA | 25 | 2.1 | 24 | 1 | 4 |
| 4 | SPAIN | 22 | 1.9 | 16 | 6 | 27.3 |
| 5 | GERMANY | 12 | 1 | 9 | 3 | 25 |
| 6 | ITALY | 12 | 1 | 9 | 3 | 25 |
| 7 | CANADA | 11 | 0.9 | 9 | 2 | 18.2 |
| 8 | FRANCE | 11 | 0.9 | 8 | 3 | 27.3 |
| 9 | INDIA | 11 | 0.9 | 8 | 3 | 27.3 |
| 10 | IRAN | 9 | 0.8 | 6 | 3 | 33.3 |
| 11 | JAPAN | 9 | 0.8 | 8 | 1 | 11.1 |
| 12 | UNITED KINGDOM | 8 | 0.7 | 4 | 4 | 50 |
| 13 | IRELAND | 5 | 0.4 | 1 | 4 | 80 |
| 14 | BELGIUM | 4 | 0.3 | 3 | 1 | 25 |
| 15 | POLAND | 4 | 0.3 | 3 | 1 | 25 |
| 16 | AUSTRALIA | 3 | 0.3 | 2 | 1 | 33.3 |
| 17 | BRAZIL | 3 | 0.3 | 2 | 1 | 33.3 |
| 18 | EGYPT | 3 | 0.3 | 2 | 1 | 33.3 |
| 19 | NETHERLANDS | 3 | 0.3 | 2 | 1 | 33.3 |
| 20 | ROMANIA | 3 | 0.3 | 3 | 0 | 0 |
| 21 | SAUDI ARABIA | 3 | 0.3 | 1 | 2 | 66.7 |
| 22 | SWEDEN | 3 | 0.3 | 2 | 1 | 33.3 |
| 23 | JAMAICA | 2 | 0.2 | 2 | 0 | 0 |
| 24 | NEW ZEALAND | 2 | 0.2 | 2 | 0 | 0 |
| 25 | UKRAINE | 2 | 0.2 | 2 | 0 | 0 |

Abbreviation: SCP: Single Country Publications; MCP: Multiple Country Publications
